# Supplementary material for: Repertoire-scale measures of antigen binding
Source: Proc Natl Acad Sci U S A. 2022 Aug 15;119(34):e2203505119. doi: 10.1073/pnas.2203505119 (PMC9407674; doi:10.1073/pnas.2203505119)
Supplement: Supplementary File [file pnas.2203505119.sapp.pdf]

Supplementary Materials, Repertoire-Scale Measures of Antigen Binding

Authors: Rohit Arora, PhD[1†] and Ramy Arnaout, MD, DPhil[1,2*]

Affiliations:

[1]Division of Clinical Pathology, Department of Pathology, Beth Israel Deaconess Medical Center, Boston, MA 02215.

[2]Division of Clinical Informatics, Department of Medicine, Beth Israel Deaconess Medical Center, Boston, MA 02215.

[†]Present address: Iktos Inc. 50 Milk Street, Floor 16, Boston, MA, 02109

*Correspondence to: rarnaout@bidmc.harvard.edu

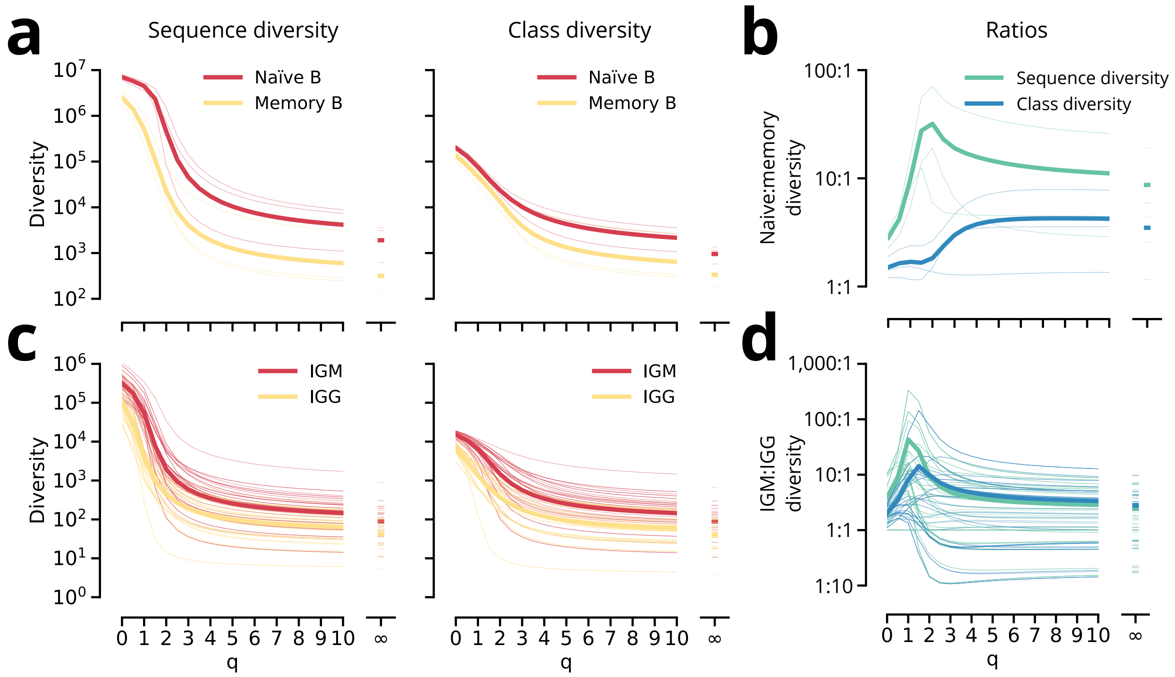

Supplementary Figure Legend

Figure S1. Sequence vs. class diversity of naive vs. memory and IGM vs. IGG repertoires. **(a)** Sequence vs. class diversity for exceptionally deeply sequenced naive vs. memory repertoires from DNA, and **(b)** the ratio of naive:memory diversity, as a function of *q* (see description of Equation 1 in the main text). Each thin line corresponds to a repertoire from a single individual. Each thick line is the average. **(c)-(d)** Analogous for IGM and IGG.
